# Predicting Early-Onset Colorectal Cancer in Individuals Below Screening Age Using Machine Learning and Real-World Data: Case Control Study

**DOI:** 10.2196/64506

**Published:** 2025-06-19

**Authors:** Chengkun Sun, Erin Mobley, Michael Quillen, Max Parker, Meghan Daly, Rui Wang, Isabela Visintin, Ziad Awad, Jennifer Fishe, Alexander Parker, Thomas George, Jiang Bian, Jie Xu

**Affiliations:** 1 Department of Health Outcomes and Biomedical Informatics, College of Medicine, University of Florida, 1889 Museum Road, Office 7020, Gainesville, FL, 32611, United States, 1 3526279467; 2 University of Florida Health Cancer Center, University of Florida, Gainesville, FL, United States; 3 Department of Surgery, University of Florida, Jacksonville, FL, United States; 4 Department of Medicine, University of Florida, Gainesville, FL, United States; 5 Center for Data Solutions, University of Florida, Jacksonville, FL, United States; 6 Department of Emergency Medicine, University of Florida, Jacksonville, FL, United States; 7 College of Medicine, University of Florida, Jacksonville, FL, United States; 8 Indiana University Indianapolis, Indianapolis, IN, United States

**Keywords:** prediction, machine learning, ML, rectal cancer, colorectal cancer, CRC, youth, adolescent, middle-aged, United States, Americans, electronic health record, EHR, Shapley Additive Explanations, SHAP, diagnosis, prevention and treatment

## Abstract

**Background:**

Colorectal cancer is now the leading cause of cancer-related deaths among young Americans. Accurate early prediction and a thorough understanding of the risk factors for early-onset colorectal cancer (EOCRC) are vital for effective prevention and treatment, particularly for patients below the recommended screening age.

**Objective:**

Our study aims to predict EOCRC using machine learning (ML) and structured electronic health record data for individuals under the screening age of 45 years, with the aim of exploring potential risk and protective factors that could support early diagnosis.

**Methods:**

We identified a cohort of patients under the age of 45 years from the OneFlorida+ Clinical Research Consortium. Given the distinct pathology of colon cancer (CC) and rectal cancer (RC), we created separate prediction models for each cancer type with various ML algorithms. We assessed multiple prediction time windows (ie, 0, 1, 3, and 5 y) and ensured robustness through propensity score matching to account for confounding variables including sex, race, ethnicity, and birth year. We conducted a comprehensive performance evaluation using metrics including area under the curve (AUC), sensitivity, specificity, positive predictive value, negative predictive value, and *F*_1_-score. Both linear (ie, logistic regression, support vector machine) and nonlinear (ie, Extreme Gradient Boosting and random forest) models were assessed to enable rigorous comparison across different classification strategies. In addition, we used the Shapley Additive Explanations to interpret the models and identify key risk and protective factors associated with EOCRC.

**Results:**

The final cohort included 1358 CC cases with 6790 matched controls, and 560 RC cases with 2800 matched controls. The RC group had a more balanced sex distribution (2:3 male-to-female) compared to the CC group (2:5 male-to-female), and both groups showed diverse racial and ethnic representation. Our predictive models demonstrated reasonable results, with AUC scores for CC prediction of 0.811, 0.748, 0.689, and 0.686 at 0, 1, 3, and 5 years before diagnosis, respectively. For RC prediction, AUC scores were 0.829, 0.771, 0.727, and 0.721 across the same time windows. Key predictive features across both cancer types included immune and digestive system disorders, secondary malignancies, and underweight status. In addition, blood diseases emerged as prominent indicators specifically for CC.

**Conclusions:**

Our findings demonstrate the potential of ML models leveraging electronic health record data to facilitate the early prediction of EOCRC in individuals under 45 years. By uncovering important risk factors and achieving promising predictive performance, this study provides preliminary insights that could inform future efforts toward earlier detection and prevention in younger populations.

## Introduction

Colorectal cancer (CRC) is a significant public health challenge, ranking as the third leading cause of cancer-related mortality among both males and females in the United States [1]. It is estimated that in 2023, approximately 153,020 individuals were diagnosed with CRC, and 52,550 succumbed to the disease [1]. While cancer is typically a disease of older age, a concerning trend has emerged—the increasing incidence of early-onset colorectal cancer (EOCRC) in individuals younger than the age of 50 years [1,2]. This increased incidence has led the US Preventive Services Task Force to modify its recommendations, lowering the age to start CRC screening to age 45 [3]. Patients diagnosed with EOCRC tend to present at later stages and face lower disease-specific survival rates, underscoring the need for early detection and treatment initiation [4]. Nevertheless, challenges in addressing EOCRC are compounded by poorly defined risk factors and the role of diagnostic delays. As a result, early prediction and comprehensive understanding of the risk factors of EOCRC are essential for prevention and treatment, particularly for patients who fall below the recommended screening age.

The rapid integration of artificial intelligence and big data analytics has significantly expanded the horizons of medical research and clinical care [5]. Diverse data sources, including imaging and genomic data, have been harnessed for CRC detection through the application of statistical and machine learning (ML) algorithms. Some approaches have included the analysis of tumor DNA and circulating RNA expression profiling data to identify potential pathogenic factors [6,7]. In addition, computer tomography (CT)–based radionics, combined with ML algorithms, have been used to predict the Kirsten rat sarcoma viral oncogene mutation in people with CRC, demonstrating the potential of ML in clinical decision support [8]. Further, a random forest (RF) model trained with standard clinical and pathological prognostic variables, coupled with magnetic resonance imaging (MRI) images, achieved an impressive area under the curve (AUC) score of 0.94 when predicting survival in CRC patients, highlighting the importance of MRI-based texture features in patient survival prediction [9]. However, imaging data produces a small number of unexplainable predictors (around 100), and does not consistently improve diagnostic accuracy and disease prediction, especially when only using imaging data [10]. Furthermore, advanced imaging modalities and genomic data can be costly, with limited accessibility, and lack diversity and representativeness in samples, which could impact timely and accurate diagnosis for all individuals affected by EOCRC or widen already present disparities in patient outcomes.

In contrast to imaging and genomic data, structured data from the electronic health record (EHR) offers a more accessible and cost-effective data source for initial research. Originally designed for administrative and billing purposes, structured EHR data have evolved into valuable tools for health care research, capturing a wealth of patient information, including clinical diagnoses, procedures, medications, and laboratory results, among others [11]. The integration of ML and deep learning with EHR data has demonstrated substantial potential for disease prediction, including Alzheimer disease, gestational diabetes mellitus, and coronary heart disease [12-14]. In the context of CRC, several ML approaches have been used to predict the risk of the disease. For example, Shanbehzadeh et al [15] used structured EHR data and four data mining algorithms to predict CRC risk, identifying critical attributes for the prediction model using the weight statistical *χ*^2^ test. However, the weight statistical *χ*^2^ test assumes independence among variables, which may not hold true in complex datasets where variables are likely correlated. Another study leveraged convolutional neural networks to predict CRC risk based on the structured EHR data from the Taiwan National Health Insurance database [16]. Hussan et al [17] explored multiple ML methods to construct predictive models for CRC among patients aged between 35 and 50 years. However, these studies faced challenges in effectively matching cases and control groups, leading to increased bias and concerns regarding confounding. Furthermore, another limitation across studies is the failure to distinguish between colon cancer (CC) and rectal cancer (RC), despite the differences in clinical presentation, molecular carcinogenesis, pathology, surgical topography and procedures, and multimodal treatment strategies between these 2 cancers [18]. In addition, the lack of model explanations regarding clinical diagnosis of CRC undermined the interpretability and reliability of their strategies. As a result, there is a pressing need for improved methodologies to enhance the reliability and understanding of ML models in EOCRC prediction.

## Methods

### Data Source and Study Population

This study used deidentified EHR data from the OneFlorida+ Clinical Research Consortium, which operates within the PCORnet Clinical Research Network funded by the Patient-Centered Outcomes Research Institute. PCORnet serves as a national resource dedicated to advancing high-priority health research and improving outcomes through a robust, integrated research infrastructure [19,20]. By combining extensive health data, research expertise, and patient perspectives, it enables network partners to rapidly generate reliable, actionable evidence to support public health and clinical decision-making [19,20]. The OneFlorida+ data encompasses a wide range of patient characteristics from health systems across the southeast, including EHR data collected using the PCORnet Common Data Model [19] regarding demographics, diagnoses, medications, procedures, vital signs, lab tests, and more.

**Figure 1.**
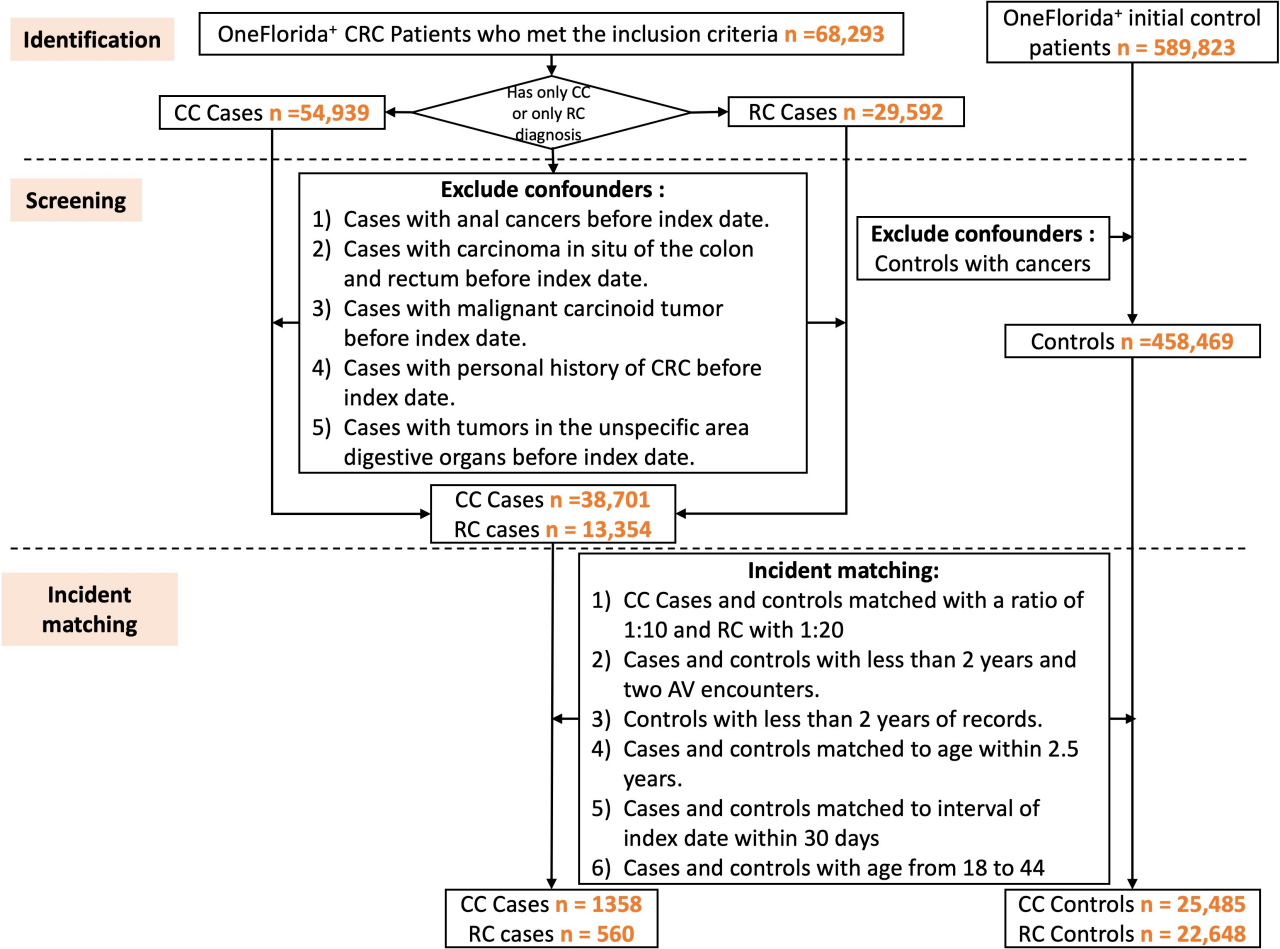
Flowchart of patient selection from OneFlorida+. CC: colon cancer; CRC: colorectal cancer; RC: rectal cancer; AV: Ambulatory Visit.

The construction of our study cohort using OneFlorida+ is outlined in Figure 1. OneFlorida+ identified individuals from the OneFlorida+ network, with encounters from January 2012 to January 2023 who met our inclusion criteria as either a case or control. We identified cases of CC using the *International Classification of Diseases, Ninth Revision* (*ICD-9*) code of C18.x or C49A4 or the *International Classification of Diseases, Tenth Revision* (*ICD-10*) code of 153.x, or RC cases with the *ICD-9* code of C19.x, C20.x, C21.0, C21.1, and *ICD-10* code of 154.0 and 154.1. The initial cohort consisted of 68,293 CRC cases (54,939 CC cases and 29,592 RC cases), and 589,823 controls. From those, we excluded patients diagnosed with both CC and RC, other previous cancers, or those who were diagnosed ≥45 years of age. Our final study cohort comprised 1358 CC cases with 25,485 controls and 560 RC cases with 22,648 controls.

We used an incident matching process to match cases and controls to ensure a fair comparison across these groups. Initially, we retained cases and controls with more than 2 years of records and at least 2 encounters before the first onset date of either CC or RC and ensured that the age gap between matched cases and controls was within 2.5 years. By calculating propensity scores based on race, ethnicity, sex, and birth year (within 2.5 y), we used a narrow caliper of 0.05 with a nearest neighbor approach to achieve a 1:5 case-to-control ratio for each prediction window group [21]. This rigorous methodology ensures a balanced study population for reliable analysis and EOCRC prediction.

### Study Setting

**Figure 2.**
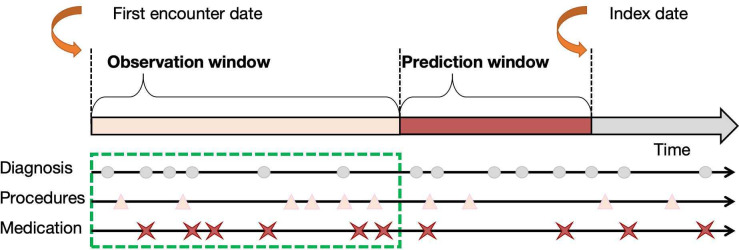
Visualization of the observation and prediction windows. For the prediction task. The index date for CRC cases is the date of diagnosis. For the control group, the index date is defined as the closest encounter date to the diagnosis date of the matched case group. The prediction window is the time period before the index date during which CRC cases are predicted. The observation window refers to the specific period during which data is collected or observed for analysis. CRC: colorectal cancer.

We then incorporated a range of different observation periods and prediction windows (Figure 2) to test our prediction algorithms, considering the different use cases. We considered 4 different prediction windows: 0 years, 1 year, 3 years, and 5 years before CRC diagnosis.

### Data Preprocessing

The predictors we extracted included data from the demographics, vitals, diagnoses, medications, and procedures tables within the OneFlorida+ Clinical Research Network throughout the observation periods. Age at index date was calculated and categorized into 3 groups (eg, 18‐29 y, 30‐39 y, and 40‐44 y). One-hot encoding [22] was used to represent age groups, race, and sex variables. Statistical analysis shows that the proportion of missing values is approximately 50% (4137/8148 in CC and 1554/3360 in RC). According to [23-25], mean imputation is less sensitive to high proportions of missing data and is more robust compared to other imputation methods, such as median and mode imputations, the indicator method, and regression. Thus, for missing data, we imputed the missing values with the mean of the numerical data derived from the entire sample within each prediction window group. Furthermore, BMI data was categorized into clinically relevant groups, including underweight (≤18.5 y), normal (18.5‐23 y), overweight (23-30 y), and obese (≥30). Diastolic and systolic measurements were categorized into distinct hypertension stages.

Diagnoses, which were initially represented using *ICD-9* and *ICD-10* codes, were subjected to a data dimensionality reduction process that mapped them into Phecodes [26,27]. Revenue codes and current procedural terminology codes [28] were leveraged to capture billed medical procedures. To integrate these data, we also used the clinical classifications software code [29]. For drug information, National Drug Code [30] and RxNorm codes were used for encoding. National Drug Codes were mapped into RxNorm codes, and further consolidated into anatomical therapeutic chemical classes [31]. To ensure completeness, all features that could not be mapped were retained to prevent any missing information. These steps to transform the data enhanced interpretability and relevance of our predictive models.

### Experiments and Validation

We explored several widely used ML models, including linear models such as logistic regression (LR) and the support vector machine (SVM), as well as nonlinear models like XGBoost (Extreme Gradient Boosting) and RF. We adopted two modeling strategies, including (1) prediction without CRC-related features and (2) prediction without cancer-related features, covering the CRC-related features. For the first strategy, features that may be indicative of CRC differential diagnoses (eg, neoplasm of unspecified nature of digestive system) or treatments for CRC (eg, chemotherapy and radiotherapy) were removed from the models and not used as predictors. For the second strategy, we took a more stringent approach by eliminating all diagnoses, drugs, and procedures that could be associated with any cancer from the extracted predictors. This step aimed to identify risk factors while eliminating the influence of other types of cancers, enabling us to focus exclusively on noncancer-related predictors. Regardless of the feature engineering strategy, we maintained a consistent experimental setup. The entire dataset was randomly split into a training dataset and a testing dataset with a ratio of 4:1. Model optimization was conducted on the training set through 5-fold cross-validation, and we fine-tuned hyperparameters using Bayesian optimization. To ensure reproducibility, we fixed the random state seed across all model runs.

To assess the effectiveness of our models comprehensively, we used a battery of evaluation metrics, including AUC, sensitivity, specificity, positive predictive value (PPV), negative predictive value (NPV), and *F*_1_-score. To mitigate the risk of overfitting and to derive robust CIs, we implemented a bootstrapping strategy. This involved conducting 100 experiments by randomly resampling the training and testing datasets. In addition to traditional performance metrics, we delved into the interpretability of the XGBoost models. Specifically, we computed Shapley Additive Explanations (SHAP) values [32] to gain insights into the inner workings of the ML algorithms and to identify the core contribution predictors. This approach aimed to unveil the high-risk factors associated with EOCRC, shedding light on the most influential features in our prediction model. To further assess generalizability, we performed temporal validation on all CC and RC groups, using data before January 1, 2015, for training and data after for testing. We then trained an XGBoost model to evaluate its performance on the test set.

### Ethical Considerations

The study was approved and the requirement to obtain any informed consent was waived by the University of Florida Institutional Review Board (protocol number IRB202201561). The research does not involve greater than minimal risk for participation. Analyses only involve the secondary analysis of data that are either limited datasets or deidentified. Our research team has no direct contact with human participants. All methods were carried out in accordance with relevant guidelines and regulations.

## Results

Table 1 provides an overview of the identified study cohorts after propensity score matching for both CC and RC across various prediction windows. Notably, CC cases outnumber RC cases, with approximately twice as many CC cases. Patients in the RC groups were slightly older compared to those in the CC group. Sex distribution in the RC groups was closer to parity (2:3 male to female) than in the CC group (2:5 male to female). Both RC and CC groups exhibited diverse racial and ethnic representation. In addition, as the prediction window lengthened, the number of cases decreased. Specifically, there were 560, 560, 383, and 225 RC cases, and 1358, 1358, 884, and 532 CC cases in prediction windows for 0 years, 1 year, 3 years, and 5 years, respectively.

**Table 1.** Descriptive statistics in case and control groups.

Variables	CC^a^ cases (n=1358)	CC controls (n=6790)	RC^b^ cases (n=560)	RC controls (n=2800)
Age, mean (SD)	36.54 (5.88)	36.69 (5.73)	37.70 (5.70)	36.80 (5.53)
Sex, n (%)				
Female	938 (69.07)	4461 (65.70)	323 (57.68)	1617 (57.75)
Male	420 (30.93)	2329 (34.30)	237 (42.32)	1183 (42.25)
Race and ethnicity, n (%)				
Hispanic	338 (24.89)	1527 (22.49)	101 (18.04)	514 (18.36)
Non-Hispanic White	554 (40.80)	2893 (42.61)	239(42.68)	1212 (43.29)
Non-Hispanic Black	353 (25.99)	1857 (27.35)	178 (31.79)	887 (31.68)
Other	14 (1.03)	66 (0.97)	4 (0.71)	9 (0.32)
Unknown	99 (7.29)	447 (6.58)	38 (6.79)	178 (6.36)
Vital Signs, missing rate, n (%)				
BMI	475 (34.98)	3393(49.97)	171 (30.54)	1383 (49.39)
Diastolic blood pressure	559 (41.16)	3578 (52.70)	236 (42.14)	1497 (53.46)
Systolic blood pressure	573 (42.19)	3618 (53.28)	240 (42.86)	1517 (54.18)
Top 10 diagnoses, n (%)				
Other tests	1016 (74.82)	5010 (73.78)	45 (8.04%)	83 (2.96)
Abdominal pain	881 (64.87)	3430 (50.52)	133 (23.75)	611 (21.82)
Other symptoms of respiratory system	630 (46.39)	2873 (42.30)	92 (16.43)	189 (6.75)
Overweight, obesity and other hyperalimentation	585 (43.08)	2915 (42.93)	46 (8.21)	187 (6.68)
Nausea and vomiting	581 (42.78)	2095 (30.85)	29 (5.18)	87 (3.11)
Nonspecific chest pain	536 (39.47)	2250 (33.14)	133 (23.75)	347(12.39)
Tobacco use disorder	523 (38.51)	2408 (35.46)	55 (9.82)	121 (4.32)
Acute upper respiratory infections of multiple or unspecified sites	522 (38.44)	2817 (41.49)	1 (0.18%)	4 (0.4)
Other anemias	512 (37.7)	1454 (21.41))	157 (28.04)	77 (2.75)
Hypertension	509 (37.48)	2202 (32.43)	127 (22.68)	557 (19.89)
Top 10 procedures, n (%)				
Other diagnostic procedures	1207 (88.88)	6241 (91.91)	63 (11.25)	100 (3.57)
Dental procedures	1071 (78.87)	5423 (79.87)	351 (62.68)	1909 (68.18)
Microscopic examination (bacterial smear; culture; toxicology)	958 (70.54)	4706 (69.31)	298 (53.21)	1917 (68.46)
Other therapeutic procedures	951 (70.03)	4720 (69.51)	296 (52.86)	1544 (55.14)
General emergency room	845 (62.22)	4097 (60.34)	203 (36.25)	622 (22.21)
Pathology	817 (60.16)	3070 (45.21)	413 (73.75)	2258 (80.64)
Chemistry laboratory-clinical	806 (59.35)	3756 (55.32)	50 (8.93)	266 (9.50)
Hematology laboratory-clinical	800 (58.91)	3740 (55.08)	470 (83.93)	2345 (83.75)
Nonoperative urinary system measurements	792 (58.32)	3784 (55.73)	138 (24.64)	763 (27.25)
General pharmacy	764 (56.26)	3556 (52.37)	47 (8.39)	229 (8.18)
Top 10 medications, n (%)				
Other analgesics and antipyretics	769 (56.63)	3301 (48.62)	5 (0.89)	0 (0.00)
Anti-inflammatory and antirheumatic products, nonsteroids	724 (53.31)	3625 (53.39)	65 (11.61)	272 (9.71)
Throat preparations	723 (53.24)	3423 (50.41)	136 (24.29)	725 (25.89)
Anti-infectives	705 (51.91)	3267 (48.11)	51 (9.11)	66 (2.36)
Opioids	687 (50.59)	2779 (40.93)	4 (0.71)	34 (1.21)
Topical products for joint and muscular pain	682 (50.22)	3427 (50.47)	1 (0.18)	1 (0.04)
Stomatological preparations	673 (49.56)	3046 (44.86)	102 (18.21)	377 (13.46)
Other gynecologicals	644 (47.42)	3283 (48.35)	80 (14.29)	361 (12.89)
Other cardiac preparations	577 (42.49)	2949 (43.43)	167 (29.82)	878 (31.36)
Corticosteroids for systemic use, plain	573 (42.19)	2520 (37.11)	121 (21.61)	528 (18.86)

a CC: colon cancer.

b RC: rectal cancer.

Table 2 presents the results of CC prediction using 2 feature engineering strategies: 1 excluding CRC-related features and the other excluding cancer-related features. Additional evaluation metrics for CC prediction across all settings can be found in Tables S1-S2 in Multimedia Appendix 1. In most cases, tree-based models (XGBoost and RF) outperformed linear models (SVM and LR), yielding higher AUC values. Specifically, after removing CRC-related features, the RF model achieved the highest AUC for the 0-year prediction (0.811, 95% CI 0.808-0.814), while RF performed best for the 1-year (0.748, 95% CI 0.745-0.751), 3-year (0.689, 95% CI 0.684-694), and 5-year (0.686, 95% CI 0.68-0.692) predictions for CC. However, after removing features associated with previous cancers, the model performance decreased: LR achieved AUC values of 0.788 (95% CI 0.786-0.791) for 0-year prediction; RF achieved AUC values of 0.716 (95% CI 0.713-0.719) for 1-year, 0.684 (95% CI 0.679-0.688) for 3-year, and 0.663 (95% CI 0.658-0.668) for 5-year prediction. Performance metrics, including specificity, sensitivity, PPV, NPV, and *F*_1_-score, exhibited similar trends.

**Table 2.** AUC^a^ comparison for colon cancer prediction using machine learning models across different prediction windows (0, 1, 3, and 5 years).

Feature strategy and model		0-year AUC (95% CI)	1-year AUC (95% CI)	3-year AUC (95% CI)	5-year AUC (95% CI)
Excluding CRC-related^b^ features					
LR^c^		0.809 (0.806-0.812)	0.733 (0.73-0.736)	0.683 (0.679-0.688)	0.674 (0.668-0.679)
SVM^d^		0.748 (0.745-0.751)	0.689 (0.685-0.692)	0.614 (0.61-0.618)	0.616 (0.61-0.621)
RF^e^		0.811 (0.808-0.814)	0.748 (0.745-0.751)	0.689 (0.684-0.694)	0.686 (0.68-0.692)
XGBoost^f^		0.802 (0.799-0.806)	0.745 (0.741-0.748)	0.689 (0.684-0.694)	0.657 (0.651-0.663)
Excluding cancer-related features					
LR		0.788 (0.786-0.791)	0.713 (0.71-0.716)	0.669 (0.665-0.674)	0.661 (0.656-0.667)
SVM		0.725 (0.722-0.729)	0.646 (0.643-0.65)	0.604 (0.6-0.608)	0.611 (0.606-0.617)
RF		0.77 (0.767-0.773)	0.716 (0.713-0.719)	0.684 (0.679-0.688)	0.663 (0.658-0.668)
XGBoost		0.76 (0.757-0.764)	0.714 (0.711-0.717)	0.662 (0.657-0.666)	0.643 (0.638-0.648)

a AUC: area under the curve.

b CRC: colorectal cancer.

c LR: logistic regression.

d SVM: support vector machine.

e RF: random forest.

f XGBoost: Extreme Gradient Boosting.

Table 3 provides RC prediction results using the same feature engineering strategies and 4 prediction windows. Additional evaluation metrics for RC prediction across all settings can be found in Tables S3-S4 in Multimedia Appendix 1. Again, after removing CRC-related features, the XGBoost model achieved the highest AUC for the 0-year prediction (0.829, 95% CI 0.825-0.834), while RF performed best for the 1-year (0.771, 95% CI 0.766-0.777), and XGBoost did best for 3-year (0.727, 95% CI 0.721-0.732), and 5-year (0.721, 95% CI 0.713-0.729) predictions for RC. Eliminating cancer-related features resulted in a performance decrease: XGBoost achieved AUC values of 0.811 (95% CI 0.806-0.815) for 0-year prediction; RF achieved AUC values of 0.756 (95% CI 0.751-0.76) for 1-year, 0.724 (95% CI 0.718-0.73) for 3-year, and 0.711 (95% CI 0.704-0.719) for 5-year predictions. Performance metrics exhibited consistent trends. In both the CC and RC prediction tasks, we observed a decline in model performance as the prediction window length increased. Notably, when we removed cancer-related features, the AUC declined. This highlights the pivotal role these features play in enhancing prediction performance.

**Table 3.** AUC^a^ comparison for rectal cancer prediction using machine learning models across different prediction windows (0, 1, 3, and 5 years).

Feature strategy and model		0-year AUC (95% CI)	1-year AUC (95% CI)	3-year AUC (95% CI)	5-year AUC (95% CI)
Excluding CRC^b^-related features					
LR^c^		0.819 (0.815-0.824)	0.763 (0.758-0.767)	0.722 (0.716-0.728)	0.693 (0.686-0.7)
SVM^d^		0.78 (0.774-0.785)	0.694 (0.689-0.699)	0.656 (0.649-0.662)	0.658 (0.65-0.665)
RF^e^		0.826 (0.822-0.83)	0.771 (0.766-0.777)	0.719 (0.713-0.726)	0.72 (0.712-0.727)
XGBoost^f^		0.829 (0.825-0.834)	0.766 (0.762-0.771)	0.727 (0.721-0.732)	0.721 (0.713-0.729)
Excluding cancer-related features					
LR		0.807 (0.803-0.812)	0.748 (0.743-0.752)	0.709 (0.703-0.715)	0.69 (0.683-0.697)
SVM		0.767 (0.761-0.772)	0.686 (0.68-0.691)	0.653 (0.646-0.659)	0.656 (0.648-0.663)
RF		0.806 (0.802-0.81)	0.756 (0.751-0.76)	0.724 (0.718-0.73)	0.711 (0.704-0.719)
XGBoost		0.811 (0.806-0.815)	0.749 (0.744-0.753)	0.724 (0.718-0.729)	0.679 (0.672-0.687)

a AUC: area under the curve.

b CRC: colorectal cancer.

c LR: logistic regression.

d SVM: support vector machine.

e RF: random forest.

f XGBoost: Extreme Gradient Boosting.

To further evaluate model performance, we integrated XGBoost and RF using soft voting. The AUC fluctuated around 0.01, showing no significant change from the best prevoting performance (Table S9 in Multimedia Appendix 1). For temporal validation, the overall AUC decreased slightly by around 0.02, suggesting potential distribution shifts over time that may have affected generalizability. While the model fit earlier data well, its weaker performance on newer data hints at possible drift (Tables S5-S8 in Multimedia Appendix 1).

**Figure 3.**
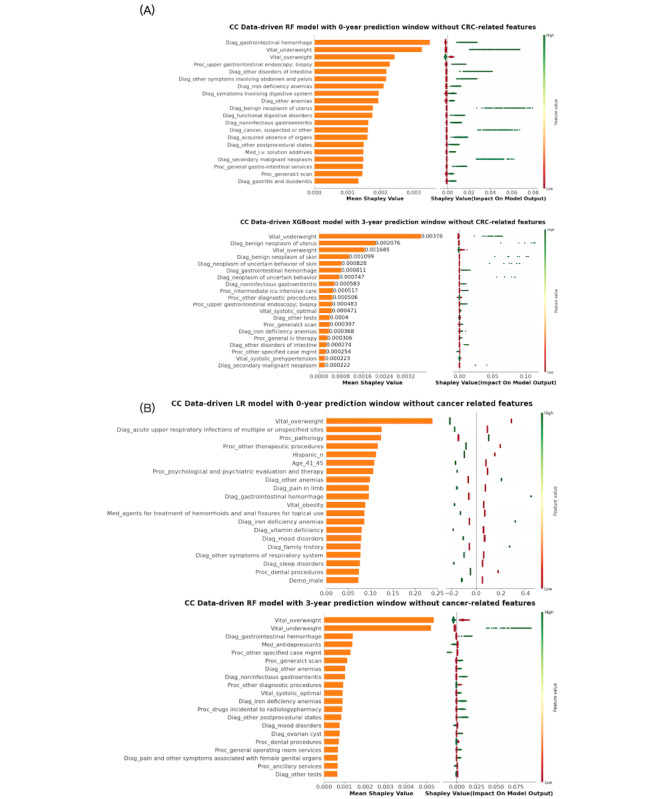
SHAP (Shapley Additive Explanations) summary plot of the top 20 features in CC prediction using best-performing models with 0-year and 3-year prediction windows: (**A**) excluding CRC-related features; (**B**) excluding cancer-related features. The prefix before the “_” in the y-axis labels of plots indicates the source of the corresponding features in the PCORnet data model. Specifically, these sources are: Diagnosis (Diag), Procedure (Proc), Medication (Med), Vital Signs (Vital), and Demographics (Demo). CC: colon cancer; CRC: colorectal cancer; LR: logistic regression; RF: random forest; XGBoost: Extreme Gradient Boosting.

To gain deeper insights into the risk factors associated with these findings, we present SHAP summary plots for CC and RC predictions using 2 feature engineering strategies and for 0-year and 3-year prediction windows in Figures3  4. Supplementary SHAP summary plots for all other models can be found in Figures S1-S2 in Multimedia Appendix 1. Within the CC group, several predictors emerged as positively associated with the risk of CC. Notably, several diagnoses involving various tumors, such as suspected cancer, secondary malignant neoplasm, benign neoplasm of uterus, benign neoplasm of skin, neoplasm of uncertain behavior, neoplasm of uncertain behavior of skin, cancer of other female genital organs and myeloproliferative diseases were identified as influential factors. Gastrointestinal symptoms, encompassing conditions like gastrointestinal hemorrhage, other disorders of intestine, other symptoms involving the abdomen and pelvis, noninfectious gastroenteritis, appendiceal conditions, diverticulosis and diverticulitis, intestinal obstruction without hernia, and disorders of the intestine also exhibited a positive association with CC risk. In addition, medical procedures related to gastrointestinal diseases and symptoms, including upper gastrointestinal endoscopy, were significantly associated with the development of CC.

**Figure 4.**
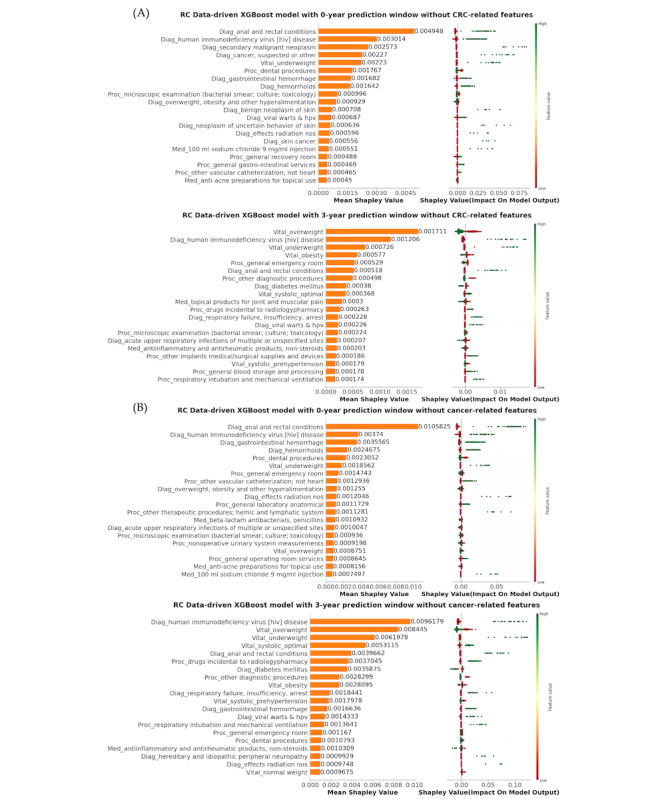
SHAP (Shapley Additive Explanations) summary plot of the top 20 features in RC prediction using best-performing models with 0-year and 3-year prediction windows: (**A**) excluding CRC-related features; (**B**) excluding cancer-related features. CRC: colorectal cancer; RC: rectal cancer; XGBoost: Extreme Gradient Boosting.

In the RC group, similar positive predictors were identified, mirroring the trends observed in the CC group, including gastrointestinal symptoms (eg, gastrointestinal hemorrhage, anal, and rectal conditions) and the presence of other cancers or tumors (eg, secondary malignant neoplasms and benign neoplasms of the uterus or skin). In addition, the presence of autoimmunity, diseases associated with a potentially weakened immune system (eg, HIV, viral warts, and human papillomavirus [HPV]), and conditions like hemorrhoids were linked to a heightened long-term risk of RC. Being underweight was a significant symptom associated with both CC and RC. Conversely, obesity, overweight, and normal weight appeared to be negatively associated with RC development. Importantly, after removing cancer-related features from consideration, the significance of anemias surged to the forefront in both the CC and RC groups. These included indicators such as iron deficiency anemias and other anemias. Nevertheless, gastrointestinal diseases and immunodeficiency pathological changes remained substantial factors contributing to CC risk, while factors such as HPV and weight retained their significance as primary determinants of RC. The use of anti-inflammatory or antirheumatic medications were associated with decreased risk of RC.

## Discussion

### Principal Findings

In this study, we used 4 traditional ML algorithms (ie, XGBoost, RF, SVM, and LR) and obtained informative results predicting EOCRC using structured EHR data. In most cases, the tree-based models, (XGBoost and RF) outperformed linear models, achieving the best AUC scores for various prediction windows. In addition, even after excluding cancer diagnosis variables (eg, pancreatic, skin, and thyroid cancer), undergoing cancer-related procedures (eg, liver biopsy and bone marrow biopsy), cancer treatments (eg, cisplatin and doxycycline), our models continued to achieve acceptable AUC scores. Immune and digestive system disorders, blood diseases, and secondary cancers were identified as significant predictors.

### Comparison to Previous Work

Most of our experimental findings were consistent with existing published research. Cancer-related diseases and diagnoses emerged as risk factors leading to the diagnosis of EOCRC, both for CC and RC. For example, uterine cancer was identified as a driver of EOCRC, suggesting a potential genetic association between these malignancies in younger patients [33]. Research also demonstrates that the incidence rate of second primary cancers among survivors is significantly higher than cancer in the general population, and survivors experience notable morbidity and mortality from their cancer treatment [34]. In addition, the use of CT scans for other medical reasons could contribute to the incidental identification of EOCRC cases [35]. Notably, we know that some forms of cancer treatment (eg, radiation) predisposes one to an increased risk for secondary malignancies, including EOCRC, particularly in patients surviving childhood cancer [36].

Inflammatory bowel diseases (IBDs) are well established risk factors for CRC, particularly during young adulthood. The chronic inflammation associated with IBD leads to the release of growth cytokines, excess blood flow, and metabolic free radicals, all of which contribute to the heightened risk of developing CRC [37]. Therapies for IBD sometimes involve immune suppression, another known risk factor for cancers. Furthermore, many gastrointestinal diseases can cause malabsorption or malnutrition [38], resulting in patients being underweight which can also contribute to immune dysfunction or suppression [39]. However, overweight patients were at low risk of EOCRC as our analysis demonstrated despite emerging evidence that being overweight may be associated with an increased risk of tumor recurrence and colorectal carcinogenesis [40,41]. The temporal use of antibiotics in relation to subsequent development of EOCRC is an interesting finding as it supports several previously reported roles that the gut microbiome may plan in CRC protection and development [42]. Our analysis highlighted that the diagnosis of iron deficiency anemia predated CC, but had less association with rectal cancers. It is logical, given that CC are situated more proximal in the gastrointestinal tract, causing occult chronic blood loss and subsequent anemia rather than overt gross bleeding as is typically evident from RC.

In addition, our study observed a significantly higher incidence of CRC cases among HIV-infected patients compared to HIV-uninfected individuals [43]. The heightened risk can be attributed to disruptions in immune function caused by immunodeficiency, which exposes individuals to a higher susceptibility against cancer-causing viruses, including HPV, Epstein–Barr virus, Kaposi sarcoma-associated herpesvirus, etc, as evidenced in our analysis [44]. Another notable finding was the association between CC and diseases of myeloproliferative disease. Similar to other cancers, the potential link could be related to genetics, treatments that induce DNA damage that could predispose to EOCRC, and chronic immune dysregulation. Overall, our study sheds light on the complex interplay between IBD, malnutrition, immune function, and specific blood-related diseases in the development of CRC. Understanding these relationships is crucial in advancing our knowledge of EOCRC risk factors and devising targeted interventions for at-risk populations. It can help health care providers identify individuals who may benefit most from screening between the ages of 18 and 44 years. In this case-control study, we identified several factors independently associated with an elevated risk of EOCRC. These findings could inform patient-provider discussions about the need for and approach to CRC screening and support targeted interventions to improve screening uptake among high-risk individuals.

### Strengths and Limitations

The strength of our study is to develop an early diagnostic tool that can help identify individuals at higher risk for EOCRC before the onset of clinical symptoms or suspicion. To further clarify, we test the algorithm across different prediction windows—0, 1, 3, and 5 years—meaning we use data from these periods before a patient’s first CRC diagnosis to predict whether they will develop CRC in the future. This approach enables us to assess how the algorithm can detect early risk signals well in advance of diagnosis, providing actionable insights for clinicians to consider for individuals who may not yet exhibit symptoms or be under suspicion for CRC. For individuals without clear clinical suspicion (ie, those who are not yet exhibiting symptoms or are below typical screening age), our algorithm could serve as a risk stratification tool. By analyzing real-world data, such as demographic information, medical history, and other relevant factors, the model can help identify patients who may benefit from earlier screening or closer monitoring, even in the absence of overt symptoms. This can be particularly important in populations with no established risk factors for CRC, but who may still be at risk for early-onset cases.

Our study does have several limitations. First, the mechanism through which identified medical factors are associated with EOCRC is speculative. For example, CT scans contributed significantly to the model’s performance, but the specific reasons are unclear. EHRs did not record the reason why patients underwent CT scans. Perhaps some patients obtained CT scans because of symptoms related to undiagnosed CRC while others received CT scans for other reasons with the incidental finding of CRC. It is less likely that CT scans could be associated with causing CRC due to radiation exposure. For that to occur, the cumulative lifetime exposure would need to be very high with exposure over a number of decades for that to occur. Perhaps CT imaging itself is just a surrogate for access to care whereby EOCRC is more likely to be eventually diagnosed as opposed to patients who might expire for other reasons with CRC, but before a diagnosis. Second, the exclusion of confounder samples and features posed difficulties, given the lack of universally accepted standards for phenotype definitions and ambiguous descriptions. These challenges hindered the design of the most optimal experiment [45]. Third, our experiments are carried out based on the EHR data, which inherently contains flaws, including missing values and potential mistakes in records. Efforts were made to fill in missing values, but comprehensive amendments remained challenging. The characteristics of the EHR data, such as temporality, irregularity, sparsity, and data imbalance, can result in abnormal outcomes when applying ML models [46,47]. Fourth, we primarily focused on metrics related to discrimination or classification (eg, AUC), as we believe these provide essential insights into how effectively the model differentiates between cases and noncases. We acknowledge that a more holistic evaluation—including calibration, fairness, stability, and net benefit—would provide a fuller picture of the model’s real-world applicability.

### Future Directions

Future research should focus on refining the experimental design, exploring alternative feature selection techniques, incorporating large language models based on both ambulatory and inpatient data, and integrating domain knowledge to enhance the performance of the prediction models. Ultimately, these efforts will contribute to early detection and better management of CRC, with the goal of improving patient outcomes. Using techniques like Synthetic Minority Over-sampling Technique or cost-sensitive learning could further improve the model’s ability to detect the minority class. These methods were not used in this study but could be considered in future work to explore their potential impact on model performance, especially in terms of improving recall for the minority class.

### Conclusion

In conclusion, our study demonstrated the potential of traditional ML algorithms in predicting EOCRC using real-world data for individuals below the screening age guideline. The identification of significant predictors and their consistency with academic research findings provide valuable insights for pursuing additional hypotheses or targeting potential patients at risk for EOCRC. However, addressing the challenges and limitations related to data quality, experimental design, and ML models’ development is essential for improving the accuracy and reliability of EOCRC prediction models.

## Supplementary material

10.2196/64506 Multimedia Appendix 1 Supplementary material.

## Abbreviations

AUC: area under the curve
CC: colon cancer
CRC: colorectal cancer
CT: computer tomography
EHR: electronic health record
EOCRC: early-onset colorectal cancer
HPV: human papillomavirus
IBD: inflammatory bowel diseases
*ICD-10*: *International Classification of Diseases, Tenth Revision*
*ICD-9*: *International Classification of Diseases, Ninth Revision*
LR: logistic regression
ML: machine learning
MRI: magnetic resonance imaging
RC: rectal cancer
RF: random forest
SHAP: Shapley Additive Explanations
SVM: support vector machine
XGBoost: Extreme Gradient Boosting
