# Supplementary material for: Predicting Early-Onset Colorectal Cancer in Individuals Below Screening Age Using Machine Learning and Real-World Data: Case Control Study
Source: JMIR Cancer. 2025 Jun 19;11:e64506. doi: 10.2196/64506 (PMC12200807; doi:10.2196/64506)
Supplement: Multimedia Appendix 1 [file cancer-v11-e64506-s001.docx]

**Table S1.** The performance of CC prediction using ML models across different prediction windows (0, 1, 3, and 5 years), excluding CRC-related features.

| **Prediction**  **Window** | **Model** | **AUC**  **(95% CI)** | **Sensitivity**  **(95% CI)** | **Specificity**  **(95% CI)** | **PPV**  **(95% CI)** | **NPV**  **(95% CI)** | **F1**  **(95% CI)** |
| --- | --- | --- | --- | --- | --- | --- | --- |
| **0-year** | LR | 0.809  (0.806,0.812) | 0.674  (0.663,0.685) | 0.807  (0.796,0.818) | 0.421  (0.41,0.433) | 0.926  (0.924,0.928) | **0.849**  **(0.847,0.851)** |
|  | SVM | 0.748  (0.745,0.751) | 0.633  (0.62,0.647) | 0.754  (0.742,0.767) | 0.347  (0.338,0.356) | 0.912  (0.91,0.914) | 0.822  (0.82,0.824) |
|  | RF | **0.811**  **(0.808,0.814)** | **0.686**  **(0.673,0.698)** | 0.791  (0.779,0.804) | 0.407  (0.396,0.418) | **0.927**  **(0.925,0.929)** | 0.78  (0.778,0.783) |
|  | XGBoost | 0.802  (0.799,0.806) | 0.61  (0.601,0.619) | **0.887**  **(0.879,0.895)** | **0.533**  **(0.518,0.548)** | 0.92  (0.918,0.921) | 0.758  (0.755,0.76) |
| **1-year** | LR | 0.733  (0.73,0.736) | 0.595  (0.581,0.609) | 0.758  (0.745,0.772) | 0.338  (0.33,0.347) | **0.904**  **(0.902,0.906)** | **0.816**  **(0.814,0.819)** |
|  | SVM | 0.689  (0.685,0.692) | 0.534  (0.514,0.554) | 0.763  (0.745,0.782) | 0.329  (0.317,0.342) | 0.893  (0.89,0.895) | 0.797  (0.795,0.799) |
|  | RF | **0.748**  **(0.745,0.751)** | **0.613**  **(0.597,0.63)** | 0.758  (0.742,0.774) | 0.351  (0.338,0.363) | 0.909  (0.906,0.911) | 0.763  (0.76,0.765) |
|  | XGBoost | 0.745  (0.741,0.748) | 0.561  (0.546,0.576) | **0.816**  **(0.801,0.831)** | **0.401**  **(0.384,0.417)** | **0.904**  **(0.902,0.906)** | 0.758  (0.755,0.76) |
| **3-year** | LR | 0.683  (0.679,0.688) | **0.574**  **(0.554,0.593)** | 0.713  (0.695,0.731) | 0.296  (0.288,0.305) | **0.894**  **(0.891,0.897)** | **0.789**  **(0.786,0.792)** |
|  | SVM | 0.614  (0.61,0.618) | 0.487  (0.466,0.508) | 0.709  (0.688,0.73) | 0.262  (0.254,0.27) | 0.874  (0.871,0.876) | 0.767  (0.765,0.77) |
|  | RF | **0.689**  **(0.684,0.694)** | 0.42  (0.405,0.435) | **0.885**  **(0.872,0.898)** | **0.45**  **(0.433,0.467)** | 0.884  (0.882,0.886) | 0.763  (0.76,0.766) |
|  | XGBoost | **0.689**  **(0.684,0.694)** | 0.455  (0.434,0.475) | 0.845  (0.825,0.864) | 0.414  (0.393,0.435) | 0.886  (0.884,0.888) | 0.757  (0.754,0.759) |
| **5-year** | LR | 0.674  (0.668,0.679) | **0.59**  **(0.565,0.616)** | 0.688  (0.664,0.712) | 0.29  (0.28,0.3) | **0.895**  **(0.892,0.899)** | **0.793**  **(0.789,0.796)** |
|  | SVM | 0.616  (0.61,0.621) | 0.57  (0.542,0.597) | 0.637  (0.61,0.664) | 0.251  (0.242,0.259) | 0.883  (0.879,0.887) | 0.767  (0.764,0.77) |
|  | RF | **0.686**  **(0.68,0.692)** | 0.552  (0.528,0.575) | 0.753  (0.732,0.775) | 0.333  (0.317,0.348) | **0.895**  **(0.892,0.898)** | 0.758  (0.754,0.761) |
|  | XGBoost | 0.657  (0.651,0.663) | 0.406  (0.388,0.425) | **0.874**  **(0.858,0.89)** | **0.436**  **(0.413,0.459)** | 0.881  (0.878,0.883) | 0.757  (0.754,0.76) |

**Table S2.**  The performance of CC prediction using ML models across different prediction windows (0, 1, 3, and 5 years), excluding cancer-related features.

| **Prediction**  **Window** | **Model** | **AUC**  **(95% CI)** | **Sensitivity**  **(95% CI)** | **Specificity**  **(95% CI)** | **PPV**  **(95% CI)** | **NPV**  **(95% CI)** | **F1**  **(95% CI)** |
| --- | --- | --- | --- | --- | --- | --- | --- |
| **0-year** | LR | **0.788**  **(0.786,0.791)** | 0.672  (0.661,0.683) | 0.777  (0.766,0.787) | 0.382  (0.373,0.391) | **0.923**  **(0.921,0.925)** | **0.829**  **(0.827,0.831)** |
|  | SVM | 0.725  (0.722,0.729) | 0.63  (0.616,0.645) | 0.725  (0.712,0.738) | 0.32  (0.312,0.327) | 0.908  (0.906,0.911) | 0.809  (0.807,0.811) |
|  | RF | 0.77  (0.767,0.773) | **0.7**  **(0.684,0.715)** | 0.71  (0.695,0.726) | 0.333  (0.325,0.342) | **0.923**  **(0.921,0.926)** | 0.758  (0.756,0.761) |
|  | XGBoost | 0.76  (0.757,0.764) | 0.595  (0.583,0.607) | **0.82**  **(0.809,0.831)** | **0.407**  **(0.397,0.417)** | 0.911  (0.909,0.913) | 0.758  (0.755,0.76) |
| **1-year** | LR | 0.713  (0.71,0.716) | 0.601  (0.587,0.616) | **0.721**  **(0.707,0.736)** | **0.307**  **(0.301,0.314)** | 0.901  (0.899,0.903) | **0.804**  **(0.801,0.806)** |
|  | SVM | 0.646  (0.643,0.65) | 0.549  (0.533,0.566) | 0.686  (0.67,0.702) | 0.263  (0.258,0.269) | 0.885  (0.882,0.887) | 0.777  (0.775,0.779) |
|  | RF | **0.716**  **(0.713,0.719)** | **0.654**  **(0.639,0.668)** | 0.671  (0.657,0.685) | 0.289  (0.282,0.296) | **0.907**  **(0.905,0.91)** | 0.758  (0.755,0.76) |
|  | XGBoost | 0.714  (0.711,0.717) | 0.614  (0.599,0.629) | 0.707  (0.691,0.722) | 0.301  (0.294,0.309) | 0.902  (0.9,0.905) | 0.758  (0.755,0.76) |
| **3-year** | LR | 0.669  (0.665,0.674) | 0.575  (0.554,0.596) | 0.69  (0.67,0.71) | 0.281  (0.273,0.29) | 0.891  (0.888,0.894) | **0.783**  **(0.78,0.786)** |
|  | SVM | 0.604  (0.6,0.608) | 0.523  (0.496,0.55) | 0.656  (0.629,0.683) | 0.246  (0.238,0.254) | 0.874  (0.871,0.877) | 0.773  (0.771,0.775) |
|  | RF | **0.684**  **(0.679,0.688)** | **0.587**  **(0.565,0.61)** | 0.692  (0.671,0.714) | 0.29  (0.279,0.3) | **0.895**  **(0.892,0.898)** | 0.764  (0.761,0.767) |
|  | XGBoost | 0.662  (0.657,0.666) | 0.494  (0.469,0.518) | **0.758**  **(0.735,0.781)** | **0.313**  **(0.3,0.325)** | 0.883  (0.88,0.886) | 0.757  (0.754,0.759) |
| **5-year** | LR | 0.661  (0.656,0.667) | **0.606**  **(0.581,0.63)** | 0.656  (0.631,0.68) | 0.272  (0.263,0.282) | **0.895**  **(0.891,0.898)** | **0.777**  **(0.773,0.78)** |
|  | SVM | 0.611  (0.606,0.617) | 0.597  (0.566,0.629) | 0.6  (0.568,0.632) | 0.242  (0.234,0.251) | 0.885  (0.881,0.889) | 0.766  (0.762,0.769) |
|  | RF | **0.663**  **(0.658,0.668**) | 0.596  (0.569,0.623) | 0.668  (0.643,0.693) | 0.281  (0.269,0.294) | 0.894  (0.891,0.898) | 0.757  (0.754,0.76) |
|  | XGBoost | 0.643  (0.638,0.648) | 0.532  (0.505,0.558) | **0.706**  **(0.68,0.731)** | **0.283**  **(0.272,0.293)** | 0.885  (0.881,0.888) | 0.757  (0.754,0.76) |

**Table S3.** The performance of RC prediction using ML models across different prediction windows (0, 1, 3, and 5 years), excluding CRC-related.

| **Prediction**  **Window** | **Model** | **AUC**  **(95% CI)** | **Sensitivity**  **(95% CI)** | **Specificity**  **(95% CI)** | **PPV**  **(95% CI)** | **NPV**  **(95% CI)** | **F1**  **(95% CI)** |
| --- | --- | --- | --- | --- | --- | --- | --- |
| **0-year** | LR | 0.819  (0.815,0.824) | 0.688  (0.674,0.701) | 0.839  (0.827,0.851) | **0.48**  **(0.462,0.498)** | 0.932  (0.929,0.934) | **0.858**  **(0.855,0.861)** |
|  | SVM | 0.78  (0.774,0.785) | 0.684  (0.672,0.696) | 0.761  (0.746,0.775) | 0.379  (0.366,0.392) | 0.927  (0.924,0.93) | 0.838  (0.835,0.841) |
|  | RF | 0.826  (0.822,0.83) | **0.7**  **(0.688,0.713)** | 0.826  (0.812,0.841) | 0.469  (0.452,0.487) | **0.934**  **(0.932,0.937)** | 0.775  (0.771,0.778) |
|  | XGBoost | **0.829**  **(0.825,0.834)** | 0.666  (0.656,0.676) | **0.877**  **(0.865,0.888)** | 0.54  (0.523,0.558) | 0.931  (0.929,0.933) | 0.758  (0.755,0.761) |
| **1-year** | LR | 0.763  (0.758,0.767) | **0.635**  **(0.619,0.65)** | 0.787  (0.773,0.802) | **0.458**  **(0.446,0.471)** | 0.894  (0.891,0.897) | 0.788  (0.784,0.792) |
|  | SVM | 0.694  (0.689,0.699) | 0.609  (0.592,0.625) | 0.705  (0.689,0.72) | 0.301  (0.293,0.308) | 0.903  (0.9,0.906) | **0.797**  **(0.794,0.8)** |
|  | RF | **0.771**  **(0.766,0.777)** | 0.583  (0.564,0.603) | 0.803  (0.784,0.823) | 0.424  (0.403,0.444) | **0.918**  **(0.915,0.92)** | 0.77  (0.767,0.773) |
|  | XGBoost | 0.766  (0.762,0.771) | 0.557  (0.539,0.574) | **0.818**  **(0.802,0.834)** | 0.432  (0.413,0.451) | 0.916  (0.914,0.918) | 0.758  (0.755,0.761) |
| **3-year** | LR | 0.722  (0.716,0.728) | **0.598**  **(0.577,0.618)** | 0.72  (0.704,0.736) | 0.382  (0.371,0.393) | 0.893  (0.888,0.897) | 0.764  (0.759,0.768) |
|  | SVM | 0.656  (0.649,0.662) | 0.545  (0.522,0.567) | 0.679  (0.654,0.705) | 0.286  (0.274,0.298) | 0.896  (0.892,0.9) | **0.786**  **(0.782,0.789)** |
|  | RF | 0.719  (0.713,0.726) | 0.525  (0.507,0.542) | 0.812  (0.794,0.83) | 0.394  (0.377,0.411) | **0.901**  **(0.898,0.904)** | 0.758  (0.754,0.762) |
|  | XGBoost | **0.727**  **(0.721,0.732)** | 0.512  (0.495,0.528) | **0.858**  **(0.839,0.877)** | **0.469**  **(0.445,0.492)** | **0.901**  **(0.898,0.904)** | 0.758  (0.754,0.762) |
| **5-year** | LR | 0.693  (0.686,0.7) | 0.616  (0.586,0.645) | 0.668  (0.64,0.697) | 0.353  (0.336,0.37) | 0.898  (0.892,0.903) | 0.767  (0.761,0.774) |
|  | SVM | 0.658  (0.65,0.665) | **0.648**  **(0.617,0.679)** | 0.647  (0.62,0.674) | 0.285  (0.274,0.295) | 0.904  (0.899,0.91) | **0.781**  **(0.776,0.786)** |
|  | RF | 0.72  (0.712,0.727) | 0.526  (0.503,0.548) | **0.766**  **(0.736,0.796)** | **0.394**  **(0.369,0.418)** | 0.91  (0.904,0.915) | 0.759  (0.754,0.764) |
|  | XGBoost | **0.721**  **(0.713,0.729)** | 0.473  (0.452,0.494) | 0.72  (0.697,0.743) | 0.343  (0.328,0.359) | **0.915**  **(0.911,0.919)** | 0.756  (0.751,0.761) |

**Table S4.** The performance of RC prediction using ML models across different prediction windows (0, 1, 3, and 5 years), excluding cancer-related features.

| **Prediction**  **Window** | **Model** | **AUC**  **(95% CI)** | **Sensitivity**  **(95% CI)** | **Specificity**  **(95% CI)** | **PPV**  **(95% CI)** | **NPV**  **(95% CI)** | **F1**  **(95% CI)** |
| --- | --- | --- | --- | --- | --- | --- | --- |
| **0-year** | LR | 0.807  (0.803,0.812) | 0.687  (0.672,0.701) | **0.821**  **(0.809,0.834)** | **0.45**  **(0.435,0.465)** | 0.93  (0.928,0.932) | **0.848**  **(0.844,0.851)** |
|  | SVM | 0.767  (0.761,0.772) | 0.684  (0.669,0.7) | 0.752  (0.738,0.767) | 0.366  (0.355,0.377) | 0.924  (0.921,0.926) | 0.829  (0.826,0.832) |
|  | RF | 0.806  (0.802,0.81) | **0.714**  **(0.701,0.727)** | 0.783  (0.77,0.795) | 0.408  (0.395,0.421) | **0.933**  **(0.93,0.935)** | 0.759  (0.756,0.762) |
|  | XGBoost | **0.811**  **(0.806,0.815)** | 0.701  (0.687,0.714) | 0.817  (0.805,0.83) | 0.448  (0.434,0.462) | **0.933**  **(0.93,0.935)** | 0.758  (0.755,0.761) |
| **1-year** | LR | 0.748  (0.743,0.752) | 0.639  (0.623,0.654) | 0.769  (0.756,0.782) | 0.366  (0.354,0.378) | 0.915  (0.912,0.918) | **0.804**  **(0.801,0.808)** |
|  | SVM | 0.686  (0.68,0.691) | 0.602  (0.583,0.621) | 0.713  (0.695,0.73) | 0.303  (0.295,0.312) | 0.901  (0.898,0.904) | 0.793  (0.791,0.796) |
|  | RF | **0.756**  **(0.751,0.76)** | **0.642**  **(0.627,0.656)** | **0.778**  **(0.765,0.791)** | 0.376  (0.365,0.387) | **0.917**  **(0.914,0.919)** | 0.758  (0.755,0.761) |
|  | XGBoost | 0.749  (0.744,0.753) | 0.623  (0.606,0.641) | **0.778**  **(0.761,0.795)** | **0.378**  **(0.363,0.394)** | 0.913  (0.91,0.916) | 0.758  (0.755,0.761) |
| **3-year** | LR | 0.709  (0.703,0.715) | **0.626**  **(0.607,0.644)** | 0.723  (0.704,0.741) | 0.323  (0.312,0.335) | 0.907  (0.904,0.91) | **0.789**  **(0.785,0.793)** |
|  | SVM | 0.653  (0.646,0.659) | 0.586  (0.558,0.613) | 0.684  (0.658,0.71) | 0.286  (0.275,0.298) | 0.894  (0.89,0.898) | 0.785  (0.782,0.789) |
|  | RF | **0.724**  **(0.718,0.73)** | 0.567  (0.548,0.586) | **0.804**  **(0.786,0.821)** | **0.385**  **(0.371,0.4)** | 0.904  (0.9,0.907) | 0.758  (0.754,0.762) |
|  | XGBoost | **0.724**  **(0.718,0.729)** | 0.607  (0.581,0.633) | 0.756  (0.731,0.782) | 0.369  (0.349,0.389) | **0.908**  **(0.904,0.912)** | 0.758  (0.754,0.762) |
| **5-year** | LR | 0.69  (0.683,0.697) | 0.631  (0.604,0.659) | 0.703  (0.676,0.73) | 0.326  (0.309,0.343) | 0.907  (0.903,0.911) | **0.795**  **(0.79,0.801)** |
|  | SVM | 0.656  (0.648,0.663) | 0.632  (0.602,0.661) | 0.661  (0.632,0.69) | 0.291  (0.278,0.304) | 0.903  (0.897,0.908) | 0.78  (0.775,0.785) |
|  | RF | **0.711**  **(0.704,0.719)** | **0.672**  **(0.645,0.699)** | 0.687  (0.66,0.713) | **0.325**  **(0.31,0.341)** | **0.915**  **(0.91,0.92)** | 0.756  (0.751,0.762) |
|  | XGBoost | 0.679  (0.672,0.687) | 0.61  (0.58,0.64) | **0.706**  **(0.677,0.734)** | 0.322  (0.306,0.338) | 0.903  (0.898,0.908) | 0.756  (0.751,0.761) |

Table S5.The temporal validation performance of RC prediction using ML models across different prediction windows (0, 1, 3, and 5 years), excluding cancer-related features.

| **Model** | **Prediction**  **Window** | **AUC** | **Sensitivity** | **Specificity** | **PPV** | **NPV** | **F1** |
| --- | --- | --- | --- | --- | --- | --- | --- |
| **XGBoost** | **0-year** | 0.784 | 0.656 | 0.809 | 0.417 | 0.920 | 0.750 |
|  | **1-year** | 0.726 | 0.511 | 0.839 | 0.398 | 0.892 | 0.750 |
|  | **3-year** | 0.683 | 0.420 | 0.922 | 0.537 | 0.881 | 0.743 |
|  | **5-year** | 0.671 | 0.882 | 0.432 | 0.259 | 0.943 | 0.735 |

Table S6.The temporal validation performance of CC prediction using ML models across different prediction windows (0, 1, 3, and 5 years), excluding cancer-related features.

| **Model** | **Prediction**  **Window** | **AUC** | **Sensitivity** | **Specificity** | **PPV** | **NPV** | **F1** |
| --- | --- | --- | --- | --- | --- | --- | --- |
| **XGBoost** | **0-year** | 0.753 | 0.583 | 0.816 | 0.365 | 0.916 | 0.777 |
|  | **1-year** | 0.701 | 0.596 | 0.696 | 0.261 | 0.905 | 0.777 |
|  | **3-year** | 0.618 | 0.291 | 0.905 | 0.357 | 0.876 | 0.777 |
|  | **5-year** | 0.604 | 0.634 | 0.624 | 0.241 | 0.901 | 0.769 |

Table S7.The temporal validation performance of RC prediction using ML models across different prediction windows (0, 1, 3, and 5 years), excluding CRC-related features.

| **Model** | **Prediction**  **Window** | **AUC** | **Sensitivity** | **Specificity** | **PPV** | **NPV** | **F1** |
| --- | --- | --- | --- | --- | --- | --- | --- |
| **XGBoost** | **0-year** | 0.806 | 0.589 | 0.894 | 0.535 | 0.913 | 0.750 |
|  | **1-year** | 0.747 | 0.440 | 0.923 | 0.559 | 0.889 | 0.750 |
|  | **3-year** | 0.662 | 0.378 | 0.972 | 0.743 | 0.879 | 0.743 |
|  | **5-year** | 0.817 | 0.765 | 0.803 | 0.464 | 0.938 | 0.735 |

Table S8.The temporal validation performance of CC prediction using ML models across different prediction windows (0, 1, 3, and 5 years), excluding CRC-related features.

| **Model** | **Prediction**  **Window** | **AUC** | **Sensitivity** | **Specificity** | **PPV** | **NPV** | **F1** |
| --- | --- | --- | --- | --- | --- | --- | --- |
| **XGBoost** | **0-year** | 0.808 | 0.642 | 0.868 | 0.467 | 0.931 | 0.777 |
|  | **1-year** | 0.715 | 0476 | 0.857 | 0.375 | 0.900 | 0.777 |
|  | **3-year** | 0.684 | 0.326 | 0.940 | 0.495 | 0.886 | 0.777 |
|  | **5-year** | 0.678 | 0.902 | 0.385 | 0.216 | 0.955 | 0.769 |

**Table S9.** The performance of soft voting strategy using RF and XGBoot models across different prediction windows (0, 1, 3, and 5 years).

|  | **Prediction**  **Window** | **AUC**  **(95% CI)** | **Sensitivity**  **(95% CI)** | **Specificity**  **(95% CI)** | **PPV**  **(95% CI)** | **NPV**  **(95% CI)** | **F1**  **(95% CI)** |
| --- | --- | --- | --- | --- | --- | --- | --- |
| **CC_ncrc** | **0-year** | 0.811  (0.809,0.814) | 0.681  (0.669,0.694) | 0.796  (0.784,0.808) | 0.41  (0.4,0.421) | 0.927  (0.925,0.929) | 0.758  (0.756,0.761) |
|  | **1-year** | 0.75  (0.747,0.753) | 0.608  (0.592,0.624) | 0.765  (0.75,0.781) | 0.355  (0.343,0.368) | 0.908  (0.906,0.91) | 0.758  (0.755,0.76) |
|  | **3-year** | 0.689  (0.684,0.694) | 0.423  (0.407,0.439) | 0.882  (0.868,0.895) | 0.447  (0.429,0.464) | 0.884  (0.882,0.886) | 0.757  (0.754,0.759) |
|  | **5-year** | 0.686  (0.68,0.692) | 0.552  (0.528,0.575) | 0.753  (0.732,0.775) | 0.333  (0.317,0.348) | 0.895  (0.892,0.898) | 0.757  (0.754,0.76) |
| **CC_nc** | **0-year** | 0.772  (0.769,0.775) | 0.681  (0.664,0.697) | 0.731  (0.714,0.747) | 0.346  (0.336,0.355) | 0.921  (0.919,0.924) | 0.758  (0.755,0.76) |
|  | **1-year** | 0.719  (0.716,0.722) | 0.66  (0.645,0.676) | 0.668  (0.653,0.683) | 0.29  (0.282,0.298) | 0.909  (0.906,0.911) | 0.758  (0.755,0.76) |
|  | **3-year** | 0.684  (0.679,0.688) | 0.585  (0.562,0.607) | 0.695  (0.673,0.716) | 0.291  (0.28,0.301) | 0.894  (0.891,0.898) | 0.757  (0.754,0.759) |
|  | **5-year** | 0.662  (0.657,0.668) | 0.597  (0.57,0.624) | 0.665  (0.639,0.691) | 0.281  (0.269,0.293) | 0.894  (0.891,0.898) | 0.757  (0.754,0.76) |
| **RC_ncrc** | **0-year** | 0.826  (0.822,0.83) | 0.704  (0.688,0.719) | 0.826  (0.812,0.841) | 0.469  (0.452,0.487) | 0.934  (0.932,0.937) | 0.758  (0.755,0.761) |
|  | **1-year** | 0.775  (0.77,0.78) | 0.628  (0.61,0.646) | 0.813  (0.796,0.831) | 0.432  (0.412,0.452) | 0.918  (0.915,0.92) | 0.759  (0.756,0.762) |
|  | **3-year** | 0.72  (0.713,0.726) | 0.551  (0.531,0.57) | 0.812  (0.794,0.83) | 0.394  (0.377,0.411) | 0.901  (0.898,0.904) | 0.758  (0.754,0.762) |
|  | **5-year** | 0.722  (0.714,0.729) | 0.62  (0.589,0.651) | 0.753  (0.723,0.783) | 0.385  (0.36,0.409) | 0.911  (0.906,0.917) | 0.756  (0.751,0.761) |
| **RC_nc** | **0-year** | 0.807  (0.803,0.811) | 0.716  (0.703,0.729) | 0.783  (0.77,0.796) | 0.408  (0.395,0.421) | 0.933  (0.931,0.936) | 0.758  (0.755,0.761) |
|  | **1-year** | 0.757  (0.752,0.762) | 0.64  (0.625,0.655) | 0.78  (0.767,0.793) | 0.377  (0.366,0.387) | 0.916  (0.914,0.919) | 0.758  (0.755,0.761) |
|  | **3-year** | 0.724  (0.719,0.73) | 0.566  (0.547,0.585) | 0.805  (0.787,0.823) | 0.389  (0.373,0.404) | 0.904  (0.9,0.907) | 0.758  (0.754,0.762) |
|  | **5-year** | 0.712  (0.705,0.72) | 0.671  (0.645,0.696) | 0.689  (0.664,0.714) | 0.325  (0.309,0.341) | 0.915  (0.91,0.919) | 0.756  (0.751,0.761) |


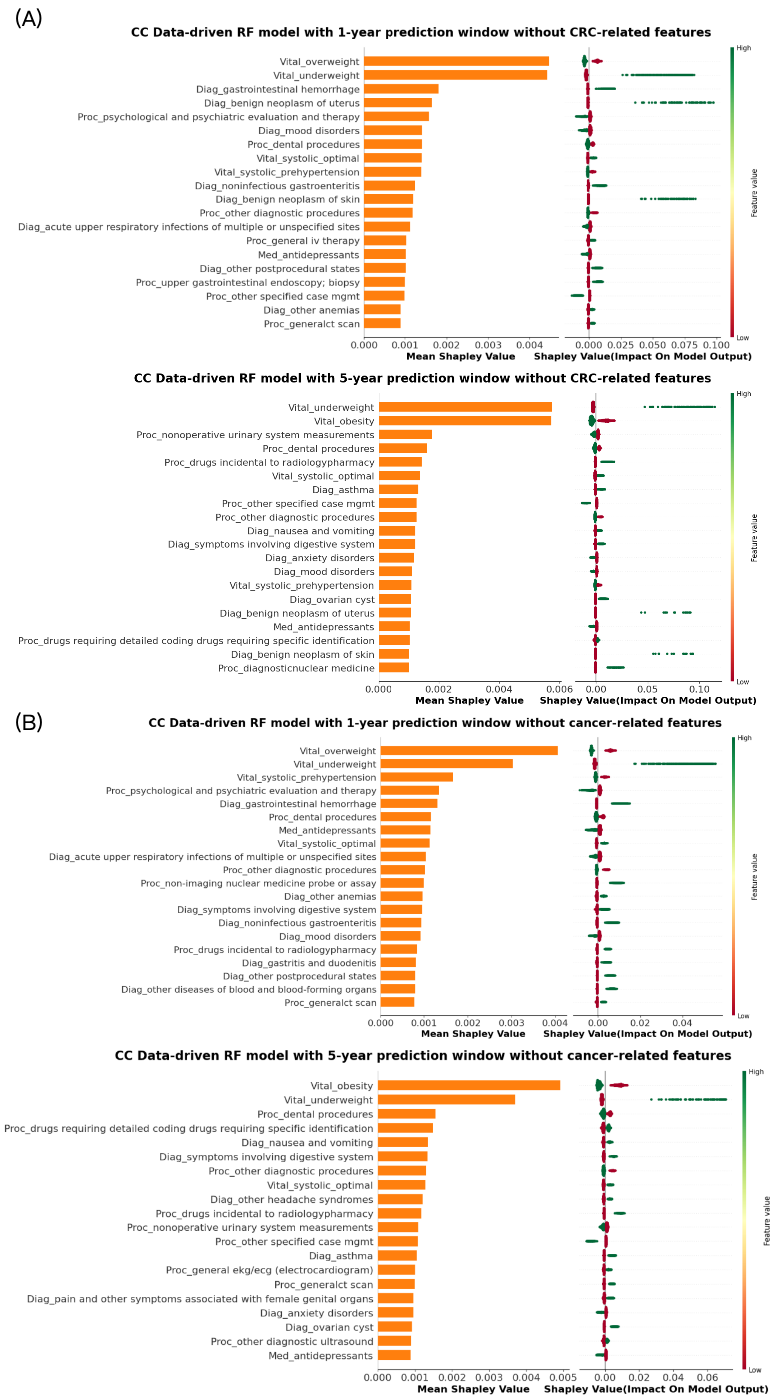


**Figure S1.** SHAP summary plot of the top 20 features in CC prediction using XGBoost models with 1-year and 5-year prediction windows: (A) excluding CRC-related features; (B) excluding cancer-related features. The prefix before the “_” in the y-axis labels of plots indicates the source of the corresponding features in the PCORnet data model. Specifically, these sources are: Diagnosis (Diag), Procedure (Proc), Medication (Med), Vital Signs (Vital), and Demographics (Demo).


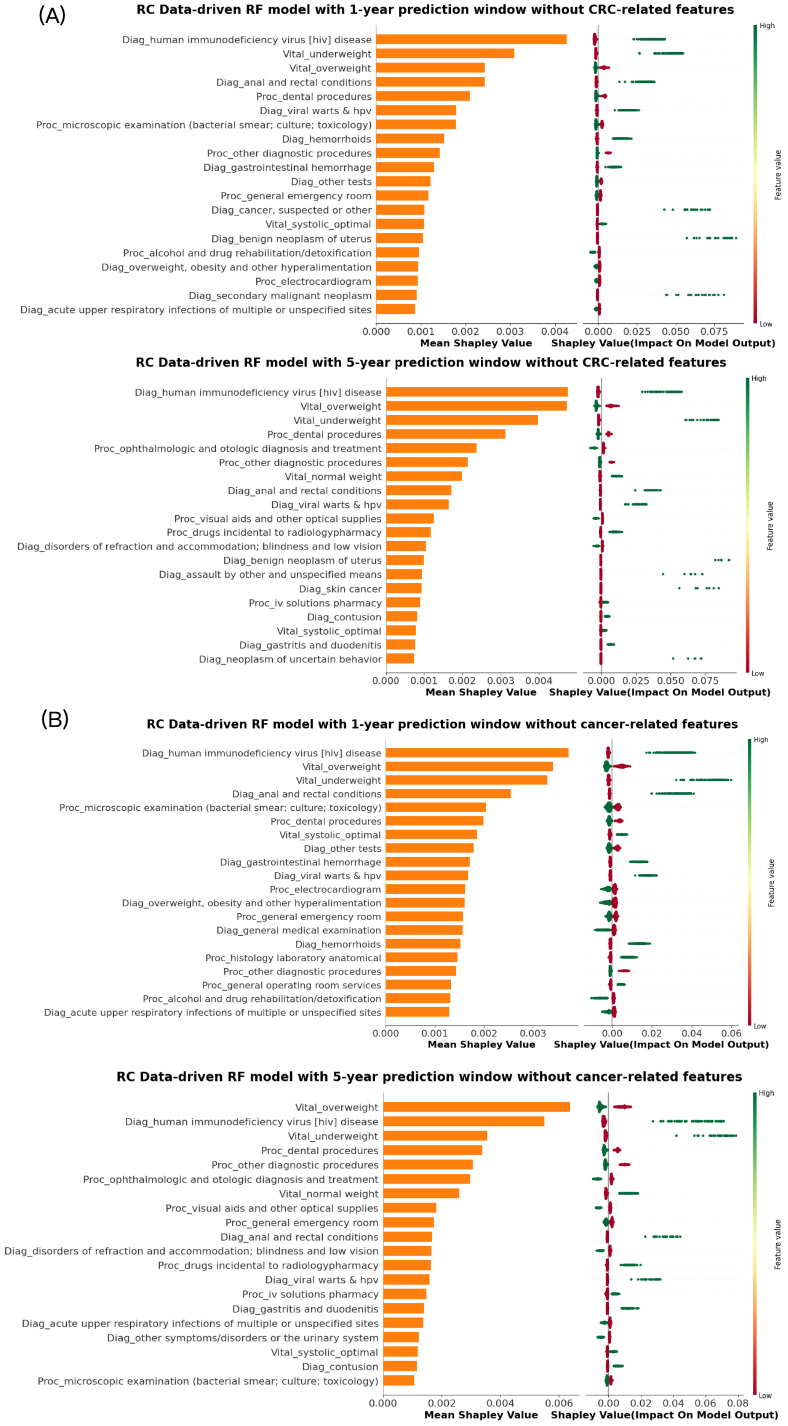


**Figure S2.** SHAP summary plot of the top 20 features in RC prediction using XGBoost models with 1-year and 5-year prediction windows: (A) excluding CRC-related features; (B) excluding cancer-related features.
